# Case of a Dolor Faciei, Where the Dissection of the Nerve Had No Effect

**Published:** 1806-03

**Authors:** 

**Affiliations:** Bayreuth


### Case of a Dolor Faciei, where the Dissection of the Nerve had no Effect. *By* Dr. Kopp, *of Bayreuth.*

A Disease whose nature and cure in general are very problematical, deserves the attention of every professional man, particularly when the efficacy of an operation, generally believed infallible, is called in question.

A lady, sixty years old, and mother of several healthy children, became affected with the dolor faciei in a most excruciating manner. The seat of the most violent pain was between the eye, nose and mouth, which increased periodically during several years. Besides these affections, she complained of a luminous shining in the eye, like lightening, when the pain commenced; which made it still more insupportable. She had never been affected with the gout or rheumatism; neither had she ever sustained a lesion of the head; and no vestige of a cancerous disposition was to be perceived. The lady had taken with great patience an immense number of medicines; but neither hemlock, nor antimony, belladonna, &c. produced the smallest relief. A great many empirical methods had been tried without success. The only hope rested on the operation, the dissection of the infraorbital nerve. About three quarters of an inch below the lower margin of the orbita, the incision was made obliquely, an inch long, and quite to the bone. The ends of the infraorbital nerve, by moving the instrument, were perfectly separated. In the same manner, about half an inch from the upper margin of the orbita, an incision was made through the *nervus frontalis.* Both wounds were kept in suppuration for some time. The operation being performed, a sedative and strengthening medicine was prescribed. Thirty-six hours after, part of the nose, and the upper lip at the left side, became cold and senseless; in the same manner the front towards the temples. The suppuration went on well. The third day after the the operation the pain commenced again, but much diminished. The luminous sensation was not perceived, and she felt no pain in the teeth of the upper jaw-bone; but her hopes soon vanished, and five months after, she suffered as much as before, requesting again the assistance of Dr. Kopp.

